# Obesity as a disease: a pressing need for alignment

**DOI:** 10.1038/s41366-024-01582-8

**Published:** 2024-07-10

**Authors:** Andrew Grannell, Carel le Roux

**Affiliations:** 1 Sidekick Health, Research & Development Unit, Kópavogur, Iceland; 2 Diabetes Complications Research Centre, University College Dublin, Dublin, Ireland

**Keywords:** Obesity, Body mass index

Obesity as a chronic disease, while generally accepted in healthcare, remains a contentious subject in the public. The pathway towards health equity from a treatment standpoint for those living with obesity starts with a scientific and widely agreed upon understanding of what the disease is and is not. As of 2024, obesity as a disease is considered “abnormal or excessive fat accumulation that presents a risk to health”, although subtle differences in definitions occur across organizations [1]. In the context of patient level care, a glaringly simple and obvious logical inconsistency is the therapeutic area remains anchored around body mass index (BMI). BMI has served a useful purpose as a crude estimate of human fat mass, allowing for valuable insights into global trends and correlations with health outcomes over time [2, 3]. However, the legacy effect of framing obesity as a disease based on BMI is holding back efforts to align society on the reality of the disease pathophysiology, leading to unintentional iatrogenesis and hindering research efforts.

Drawing out this logical inconsistency is the fact that abnormal or excessive fat accumulation that presents a risk to health occurs at low BMIs. This has been documented in people with hypertension and a BMI < 25 kg/m^2^ but the presence of visceral adiposity [4]. In addition, many people with “normal weight” develop type 2 diabetes (T2D). As demonstrated in the ReTUNE study, for these “normal weight” individuals intentional weight loss brings the disease under control mechanistically through reductions in intra-hepatic fat [5]. This is a clear indication that the pathophysiology T2D in these patients relates in part to excess adipose tissue and thus excess overall body weight or in some, simply fat in the wrong places such as pancreatic steatosis. This is clearly obesity, or perhaps a specific sub-type. To add more complexity, it is not difficult to imagine a “normal weight” patient with obesity characterized by fat in the wrong places, whose overall body weight does not change with successful treatment. In this case, the reductions in muscle, liver, and pancreatic fat can occur concurrently with an increase in skeletal muscle mass.

Obesity as a disease appears to present with signs, symptoms and characteristics from an appetitive, inflammatory and mechanical standpoint [6–8]. Breakthroughs in glucagon-like peptide-1 pharmacotherapies and insights into the mechanism of action of bariatric surgery point strongly towards appetitive aspects playing a central role in disease pathophysiology. However, we must acknowledge the reality that some people living in a bigger body do not have these characteristics, signs or symptoms and do not develop health complications associated with their weight. Therefore, it is clear that beyond epidemiological research, where BMI serves a useful purpose, obesity as a disease at the patient level should not be attached to BMI. With this in mind it is a serious problem if we do not address this issue. From a public health standpoint, the reality is that many people living with obesity will remain undiagnosed and untreated. At the same time, many people will unnecessarily be treated for a pathophysiology that is simply not present.

And where people do have the disease of obesity, the treatment targets will remain misplaced. Despite many healthcare professionals viewing obesity as a disease they still refer to concepts of normal weight and overweight, which are logically inconsistent in light of the reality of the pathophysiology occurring in a range of body sizes. A result of not acknowledging this reality is that, unintentionally, prevention efforts will be hindered while at the same time, stigma and weight bias will continue unabated. While for many, their disease is visible, for others, it is not. Therefore, assuming someone living in a bigger body is a sick person is false. Thus, the negative stereotypes that have existed for centuries and infiltrated all aspects of modern society are, in fact, based on a fallacy. The message that obesity is not your fault is clear, but at the same time, a bigger body size is not a fault.

The implications of thinking beyond BMI, as described above, are significant from a treatment standpoint, but just as important are research considerations. Treatment criteria are based on inclusion criteria for clinical trials, which remain anchored around BMI. The same goes for genome-wide associated studies and subsequent efforts to develop gene therapies [9]. If obesity is indeed characterized by diverse signs, symptoms and characteristics occurring across a range of body sizes, new ways of thinking are needed. The generation of new hypotheses from the perspective of understanding obesity requires going beyond the traditional energy balance model [10]. While this model helps shed light on the causes of obesity it does not explain the pathophysiology that represents the disease itself. A unified theory of what obesity is may require the integration additional models and concepts such as the dual intervention point model, the personal fat threshold concept and the Leeds model of appetite regulation [5, 11, 12]. From the perspective of understanding obesity as a broad set of diseases the dual intervention point model may allow us to understand the genes and potential pathophysiology responsible for heterogenous weak defense against weight gain and strong defense against weight loss across a range of body sizes. Concurrently the Leeds model of appetite can allow us ask questions regarding the presence or absence of appetite dysregulation in individuals with obesity with opposing body sizes on the BMI spectrum as unintentional weight gain and weight regain are occurring. And importantly the personal fat threshold concept may shine light on the emergence of pathophysiology and health complications occurring across a range of body sizes.

Elucidating potential sub-types of obesity beyond BMI can allow us to begin conducting research into the utility of lifestyle, pharmacotherapy and surgery for specific cases. It is only with a deeper understanding of obesity beyond BMI that novel techniques such as gene editing and optogenetics can truly be explored [13]. Thus, from an assessment standpoint it is clear we cannot identify that which we do not fully understand. However, with a deeper understand of obesity we can then develop new holistic assessment tools that go beyond BMI to examine signs, symptoms and characteristics across appetitive, inflammatory and mechanical domains. In addition technologies that serve as non-invasive means of examining where an individual has gone beyond their ”personal fat threshold” can be leveraged.

However, a lot must be done for the field to align with this proposed reality we describe. Efforts are currently taking place where different expert groups attempt to develop new guidelines around specific definitions of obesity [14, 15]. This runs the risk of increasing levels of confusion within an already confused healthcare system and the general public. Perhaps we need to admit that a complex set of diseases which obesity likely is, cannot be described with a simplistic definition. We, therefore, need new research questions and new hypotheses in order to better characterize a set of diseases with diverse signs, symptoms and characteristics. We hope this perspective brings together experts across the field to stimulate collective thinking. Given the arrival of safe, effective yet dangerously potent pharmacotherapies along with artificial intelligence posed to radically change the face of healthcare where current large language models frame obesity around BMI, it is clear we need to act now. The history of medicine shows us that when light is shone on the biological determinants of disease that stigma, shame and suffering are reduced [16]. We must keep in mind effect science has had on transforming negative perceptions around epilepsy, schizophrenia, and AIDs. However, despite advancements in obesity science, negative perceptions persist. Perhaps a logically consistent and widely agreed upon definition of obesity may be the impetus for change.
